# Expression and clinical significance of NCOA5 in epithelial ovarian cancer

**DOI:** 10.3389/fonc.2023.1117033

**Published:** 2023-05-01

**Authors:** Xiaoping Song, Da Qian, Ping Dai, Qian Li, Qiuping Xi, Kailv Sun

**Affiliations:** ^1^ Department of Gynaecology, Changshu Hospital Affiliated to Soochow University, Changshu No.1 People’s Hospital, Changshu, Jiangsu, China; ^2^ Department of Burn and Plastic Surgery-Hand Surgery, Changshu Hospital Affiliated to Soochow University, Changshu No.1 People’s Hospital, Changshu, Jiangsu, China; ^3^ Department of Thyroid and Breast Surgery, Changshu Hospital Affiliated to Soochow University, Changshu No.1 People’s Hospital, Changshu, Jiangsu, China

**Keywords:** NCOA5, clinical significance, survival, progression, epithelial ovarian cancer

## Abstract

**Background:**

Nuclear receptor coactivator 5 (NCOA5) plays a significant role in the progression of human cancer. However, its expression in epithelial ovarian cancer (EOC) is unknown. The current study was designed to explore to investigate the clinical significance of NCOA5 and its correlation with the prognosis of EOC.

**Methods:**

Immunohistochemistry was used to detect the expression of NCOA5 in 60 patients with EOC in this retrospective study and statistical analysis was performed to assess its relevance to clinicopathologic features and survival.

**Results:**

NCOA5 expression was significantly higher in EOC than in normal ovarian tissues (P < 0.001). Its expression level was significantly correlated with FIGO stage (P <0. 05) and subtypes of ovarian cancer (P < 0.001), while not correlation with age , differentiation and lymph node metastasis (P>0.05). Correlation analysis showed that NCOA5 was significantly correlated with CA125 (P < 0.001) and HE4 (P < 0.01). In a Kaplan-Meier analysis of overall survival rates, the patients with low expression of NCOA5 had significantly longer survival than high expression of NCOA5 (p=0.038).

**Conclusion:**

NCOA5 high expression is associated with EOC progression and can be an independent factor affecting the prognosis of EOC patients.

## Introduction

Ovarian cancer is the seventh most common cancer among women (1). Approximately 90% of ovarian cancers are EOC (2). A total of 23,000 EOC deaths were reported, the crude mortality rate was 3.37/100,000 in females in China (3). Cytoreductive surgery and chemotherapy are the main treatments for ovarian cancer (4). The prognosis and overall survival of ovarian cancer still remains poor due to late diagnosis and chemotherapy resistance (5, 6). Effective management of epithelial ovarian cancer (EOC) requires a multidisciplinary approach from various disciplines. The multidisciplinary treatment plan may include surgery, chemotherapy, and radiation therapy, among other interventions. Multidisciplinary management of EOC patients is essential for improving the probability of a successful outcome (7). Besides, Goal of multidisciplinary treatment is to not only treat the cancer but also to manage the symptoms of patients and improve their quality of life. The molecular mechanisms of ovarian cancer have not been elucidated. Therefore, highlighting the importance of identifying molecular mechanisms that would improve the prognosis of ovarian cancer is urgently required.

The nuclear receptor coactivator 5(NCOA5), also known as coactivator independent of AF-2, can enhance ER transcription activity (8). Previous studies (9, 10) revealed that NCOA5 insufficiency increases the risk of both glucose intolerance and inflammatory phenotype, resulting in the development of hepatocellular carcinoma (HCC). Identical, NCOA5 low expression is associated with esophageal squamous cell carcinoma (11).However, previous studies have already demonstrated that NCOA5 was involved in tumorigenesis in several types of cancer including breast cancer (12) and colorectal cancer (13). In the present study, we investigated roles of NCOA5 in epithelial ovarian carcinoma.

## Materials and methods

### Patients and samples

In this retrospective study, a total of 60 specimens paraffin sections were obtained from EOC patients who have undergone cytoreductive surgery in the Changshu No.1 People’s Hospital from March 2013 to May 2018 for Immunohistochemistry analysis. Meanwhile, twenty specimens paraffin sections of normal ovarian tissues were resected because of uterine or fallopian tube surgery. Pathological staging was reviewed independently by two experienced pathologists according to the 7th edition of the American Joint Committee’s Cancer Staging Manual (14). Clinical information was gathered from the patient’s records retrospectively. This study was approved in writing by Changshu No.1 People’s Hospital’s ethics committee(No.87).

### Immunohistochemistry analysis

This study used the method of Sun et al. (12) and the methods description partly reproduced their wording. Formalin-fixed and paraffin-embedded EOC tissues and normal ovarian tissues were cut into 4 μm thick sections. The sections were then deparaffinized and rehydrated. Antigen retrieval was performed by microaving the slides in 0.01 M citrate buffer (pH=6.0) for a total of 10 min. Endogenous peroxidase activity was quenched by treatment with 3% H2O2 for 30 min followed by incubation with goat serum for 15 min. Subsequently, the sections were incubated with rabbit anti-human NCOA5 (1:25; Cat. No.A300-789A, Bethyl, Montgomery, TX, USA) primary antibody in a humidity chamber overnight at 4°C. The primary antibody was omitted for a negative control. Horseradish peroxidase (HRP)-labeled anti-rabbit secondary antibody was then incubated for 1 hour at room temperature and the immunostaining signal was detected using a UltraSensitiveTM SP kit (Maxin, Fuzhou, Guangdong, China). Finally, the slides were counterstained with hematoxylin & eosin (HE) and coverslipped. Immunohistochemistry scores were independently examined by two experienced histopathologists without knowledge of clinicopathological information. The percentage of positive tumor cells and the staining intensity were used to gain the immunohistochemistry scoring. The percentage of positive tumor cells was assigned to 5 categories: ≤5% (0), 5-25% (1), 25-50% (2), 50-75% (3), and ≥75% (4). Positive cells (≤5%) were used as the cut-off to define negative tumors. The intensity of immunostaining was scored as follows: negative (0), weak (1), moderate (2), and strong (3). The percentage of positive tumor cells and staining intensity were added to produce a weighted score for each tumor specimen. The intensity scores were grouped as (-), 0-1; (+), 2-3; (++), 4-5; and (+++), 6-7. It was considered as high expression in tumor specimens when the final scores were ≥4 (++, +++) (15).

### Statistical analysis

SPSS version 23.0 (SPSS Inc., Chicago, IL, USA) and GraphPad Prism version 6.01 (GraphPad Software Inc., La Jolla, CA, USA) were performed to analyze data. Measurement data were described as ± standard deviation, and comparison between groups was performed by independent sample t test or one-way analysis of variance. Pearson correlation coefficient was used to evaluate the correlation between NCOA5 and other tumor markers. Kaplan-Meier survival curve was used to analyze the overall survival (OS) and progression-free survival (PFS) of patients with different NCOA5 expression, and Log-rank test was performed. A two-tailed p-value of less than 0.05 was considered statistically significant.

## Results

### NCOA5 expression in EOC

**Figure 1:**
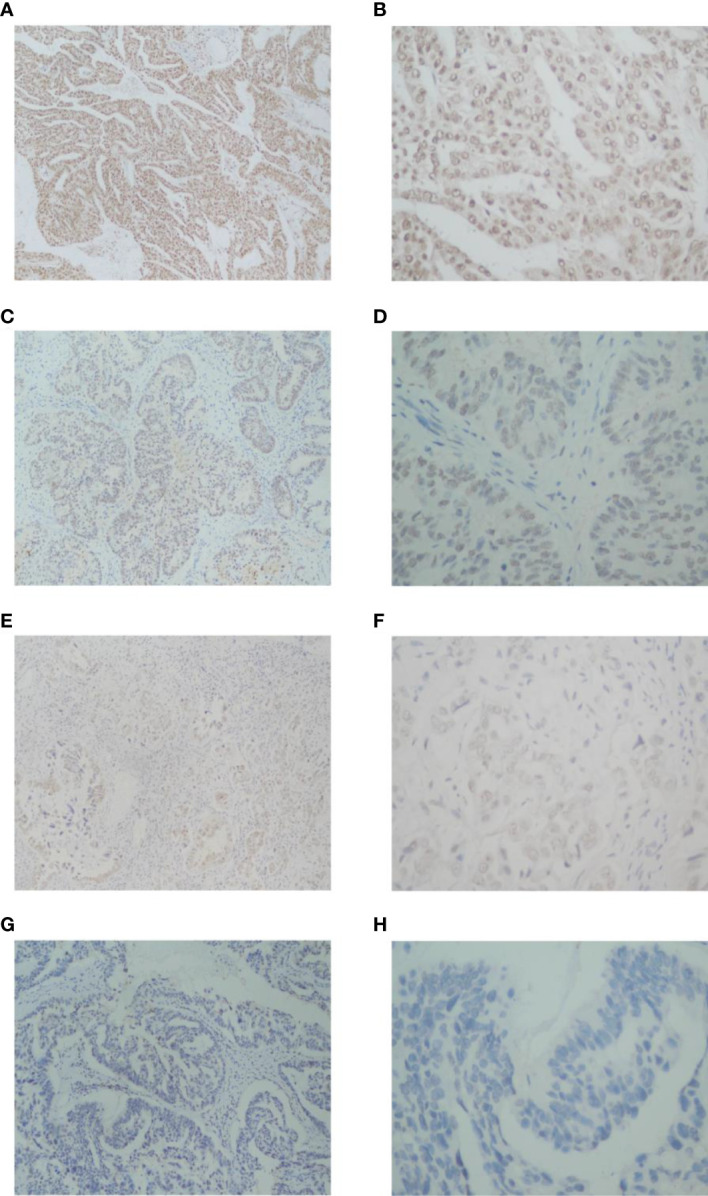
Immunohistochemistry analysis of NCOA5 in EOC tissue specimens. The representative pictures +++ (**A**: ×100, **B**: ×400),++ (**C**: ×100, **D**: ×400),+ (**E**: ×100, **F**: ×400) and -(**G**: ×100, **H**: ×400).

**Figure 2:**
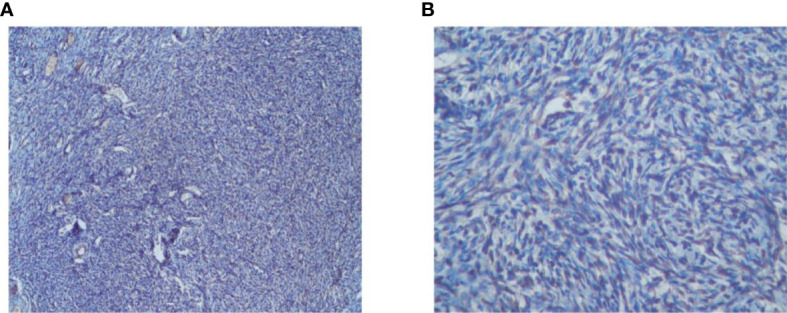
Immunohistochemistry analysis of NCOA5 in normal ovarian tissues specimens (**A**: ×100, **B**: ×400).

The study included 60 patients with clinicopathological features representative of EOC. To characterize its expression pattern in EOC, the expression of NCOA5 in human EOC tumor tissues and normal ovarian tissues was evaluated by immunohistochemistry analysis. As shown in **Figures 1A–H**, NCOA5 protein which was stained yellow brown was detected mainly in the nucleus. We found that NCOA5 expression was significantly higher in EOC than in normal ovarian tissues (**Figure 2**) (P < 0.001, **Table 1**). What calls for special attention is that the expression level of NCOA5 was significantly higher in serous ovarian cancer than in other EOC (P<0.001, **Table 2**).

**Table 1:** Comparison of NCOA5 expression between EOC and normal ovarian tissues.

Groups	Numbers	The expression level of NCOA5
EOC	60	1.38 ± 0.14
Normal ovarian tissues	20	0.67 ± 0.10
t value		61.02
P value		0.000

**Table 2:** The relationship of NCOA5 expression with EOC clinicopathological features (Pearson’s χ^2^ test).

Variables	Numbers	The expression level of NCOA5	t value	P value
Age (year)			0.895	0.314
≥50	34	0.27 ± 0.12		
<50	26	0.30 ± 0.08		
Differentiation			0.583	0.171
High	16	0.38 ± 0.10		
Moderately	21	0.29 ± 0.11		
Poorly	23	0.16 ± 0.09		
FIGO Stage			0.763	0.002
I-II	26	0.59 ± 0.07		
III-IV	34	1.02 ± 0.05		
Subtype			0.685	<0.001
Serous	40	1.97 ± 0.11		
Non- Serous	20	0.66 ± 0.03		
Lymph nodes			0.614	0.173
Positive	25	0.21 ± 0.09		
Negative	35	0.35 ± 0.12		

### Correlation between NCOA5 expression and prognosis

**Figure 3:**
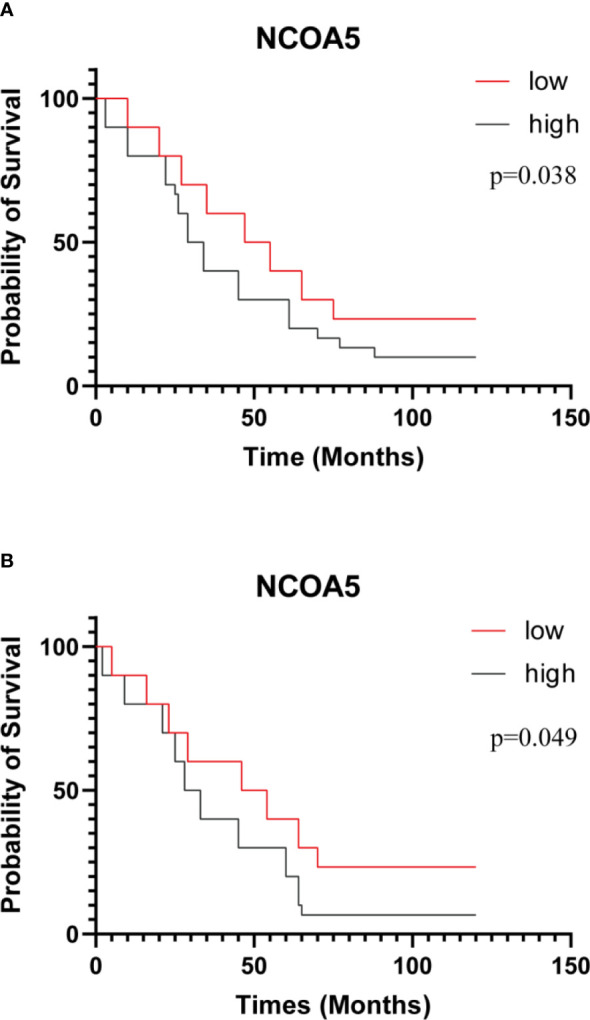
Kaplan-Meier curves of OS **(A)** and PFS **(B)** after surgery for all patients. patients with low expression of NCOA5 had significantly longer survival than high expression of NCOA5 (p=0.038、p=0.049).

There was a significant difference in the expression of NCOA5 in FIGO stage of ovarian cancer patients (P<0.001), but there were no significant differences with age, Differentiation degree and lymph node metastasis(P>0.05, **Table 2**). The association between NCOA5 and CA125 in predicting EOC was 0.719(P<0.001), meanwhile, NCOA5 and human epididymal protein 4(HE4) in predicting EOC was 0.766 (P=0.001, **Table 3**). OS and PFS for patients after surgery with low expression of NCOA5 had significantly longer survival than high expression of NCOA5(p=0.038, p=0.049) (**Figure 3**).

**Table 3:** Correlation analysis between NCOA5 expression and other tumor markers.

NCOA5	CA125	HE4	Pre-ROMA	Post-ROMA
r	0.719	0.766	0.711	0.802
P	<0.001	0.001	0.032	0.062

## Discussion

Previous researches revealed that NCOA5 expression were related to breast cancer (12) and colorectal cancer (13). Conversely, It has also been reported that NCOA5 expression is downregulated in hepatocellular carcinoma (9), esophageal squamous cell carcinoma (11), papillary thyroid carcinoma (16), and cervical cancer (17). However, the potential correlation between NCOA5 and clinical outcome in patients with EOC has not been reported. In the present study, NCOA5 expression was significantly increased in EOC compared with normal ovarian tissues, it suggests that NCOA5 is probably involved in the carcinogenesis of EOC.

CA125 has been an established protein marker for the detection and monitoring of ovarian cancer therapy for many years. Since 2003, the increase of serum HE4 level has attracted increasingly attention for predicting ovarian cancer. In addition to finding specific and sensitive markers, ROMA algorithm was developed (18). In our study, the expression of NCOA5 was highly correlated with CA125 and HE4. As suggested by the results above, NCOA5 may meet the criteria of a good diagnostic test for EOC. It is worth noting that the correlation between NCOA5 and pre-Roma is better than that between post-Roma. However, due to the small number of cases, observation after expanding the number of cases may be necessary.

Tan et al. (19) reported that knockdown of NCOA5 in breast cancer cells significantly decreased the expression levels of N-cadherin and Vimentin, whereas increased the expression levels of E-cadherin, on the contrary, upregulation of N-cadherin and Vimentin expression or downregulation of E-cadherin expression contribute to epithelial-mesenchymal transition (20). The expression level of NCOA5 in FIGO stage III/IV was significantly higher than that in FIGO stage I/II patients, indicating that NOCA5 may play a role in the distant metastasis of ovarian cancer, however, it needs to be verified by cell experiments.

This study provides new insights and evidences that NCOA5 is significantly correlated with progression and prognosis in EOC. The expression of NCOA5 in ovarian cancer and its role in tumor development remain unclear. Due to heterogeneity between different databases, differences between study populations NCOA5 is not listed as a prognostic marker in ovarian cancer at present in protein atlas database. Our study reveals that NCOA5 has great potential as a novel prognostic marker in ovarian cancer. There are several limitations of this study have to be pointed out. Firstly, in the present study, we only included a small sample size in this single-center study, whether the research conclusion is universal will be verified by accumulating more subjects in the future work. Secondly, several clinical characteristics of enrolled patients were heterogeneous, moreover, we did not study the prognostic values of NCOA5 in patients regarding to the stage of the EOC. Finally, retrospective studies have several inherent biases, including selection bias, recall bias, reporting bias and confounding bias which can affect the reliability of the findings.

NCOA5 plays a critical role in the occurrence and development of several cancers by regulating various cellular processes such as cell proliferation, differentiation, and apoptosis. NCOA5 expression is downregulated in breast cancer cells, which promotes the proliferation and survival of cancer cells (19). Moreover, NCOA5 has been found to suppress the expression of cancer stem cell markers, thereby inhibiting tumor growth and metastasis. Moreover, NCOA5 has been shown to suppress the growth and metastasis of colorectal cancer cells by inhibiting the activation of the PI3K/Akt signaling pathway (13). NCOA5 also enhances the sensitivity of colorectal cancer cells to chemotherapy drugs, thereby improving the therapeutic efficacy. Besides, NCOA5 expression is significantly decreased in hepatocellular carcinoma cells compared to normal tissues. It has been demonstrated that NCOA5 inhibits the proliferation, migration, and invasion of gastric cancer cells by suppressing the expression of matrix metalloproteinases (MMPs) (21). Therefore, NCOA5 may serve as a potential therapeutic target for the treatment of cancers. However, the association between NCOA5 and ovarian cancer and its underlying mechanisms has not been reported yet. The overexpression of NOCA5 in patients with advanced stages of ovarian cancer suggests that this gene may play a crucial role in promoting distant metastasis. There are several potential mechanisms by which NOCA5 could contribute to the metastatic spread of ovarian cancer cells. Firstly, NOCA5 may regulate the activity of specific genes involved in the metastatic process, such as genes involved in cell migration, invasion, and adhesion. For instance, NOCA5 may enhance the expression of genes that promote the formation of new blood vessels, which is essential for the growth of metastatic tumors in distant organs. Secondly, NOCA5 could promote the epithelial-mesenchymal transition (EMT), which is a cellular process that allows cancer cells to acquire a more invasive phenotype. During EMT, cancer cells lose their cell-cell adhesion properties and gain a mesenchymal-like phenotype, which allows them to migrate through tissues and invade blood vessels (21). Thirdly, NOCA5 may also be involved in the regulation of the tumor microenvironment, which can play a critical role in promoting tumor growth and metastasis. For instance, NOCA5 could stimulate the production of cytokines and growth factors that promote angiogenesis and suppress immune surveillance (22). Molecular parameters such as genetic mutations, gene expression profiles, and protein levels can provide valuable information on the biology of ovarian cancer and its response to therapy. The combination of molecular targeting treatment with classical medical imaging methods such as positron emission tomography/computed tomography (PET/CT) can potentially lead to the development of better early diagnosis models and will improve the management of patients with EOC (7, 23, 24). We suggest that NCOA5 gene may have potential value as a pathological diagnostic marker of ovarian cancer. NCOA5 was highly expressed in serous ovarian cancer and closely related to CA125 and HE4. NCOA5 may be a promising supplementary marker in serous ovarian cancer and help determine its management in the future. However, basic experiments are still needed to further confirm the role of NCOA5 in the development of EOC.

## Data availability statement

The original contributions presented in the study are included in the article/supplementary material. Further inquiries can be directed to the corresponding authors.

## Ethics statement

The studies involving human participants were reviewed and approved by Clinical ethical Committee of Changshu No.1 People’s Hospital. The patients/participants provided their written informed consent to participate in this study.

## Author contributions

XS designed research. XS, DQ, PD, QL, and QX carried out research. DQ and KS analyzed data. XS, DQ, and KS drawn the figures and made the tables. XS, DQ, and PD wrote and revised the manuscript. All authors contributed to the article and approved the submitted version.

## References

[1] Momenimovahed Z Tiznobaik A Taheri S Salehiniya H. Ovarian cancer in the world: epidemiology and risk factors. Int J Womens Health (2019) 11:287–99. doi: 10.2147/IJWH.S197604 PMC6500433 31118829

[2] Torre LA Trabert B DeSantis CE Miller KD Samimi G Runowicz CD. Ovarian cancer statistics, 2018. CA Cancer J Clin (2018) 68(4):284–96. doi: 10.3322/caac.21456 PMC6621554 29809280

[3] Chen W Sun K Zheng R Zeng H Zhang S Xia C. Cancer incidence and mortality in China, 2014. Chin J Cancer Res (2018) 30(1):1–12. doi: 10.21147/j.issn.1000-9604.2018.01.01 29545714 PMC5842223

[4] Bowtell DD Böhm S Ahmed AA Aspuria PJ Bast RC Jr Beral V. Rethinking ovarian cancer II: reducing mortality from high-grade serous ovarian cancer. Nat Rev Cancer (2015) 15(11):668–79. doi: 10.1038/nrc4019 PMC4892184 26493647

[5] Coleman RL Monk BJ Sood AK Herzog TJ. Latest research and treatment of advanced-stage epithelial ovarian cancer. Nat Rev Clin Oncol (2013) 10(4):211–24. doi: 10.1038/nrclinonc.2013.5 PMC3786558 23381004

[6] Oronsky B Ray CM Spira AI Trepel JB Carter CA Cottrill HM. A brief review of the management of platinum-resistant-platinum-refractory ovarian cancer. Med Oncol (2017) 34(6):103. doi: 10.1007/s12032-017-0960-z 28444622

[7] Delgado Bolton RC Aide N Colletti PM Ferrero A Paez D Skanjeti A. EANM guideline on the role of 2-[18F]FDG PET/CT in diagnosis, staging, prognostic value, therapy assessment and restaging of ovarian cancer, endorsed by the American college of nuclear medicine (ACNM), the society of nuclear medicine and molecular imaging (SNMMI) and the international atomic energy agency (IAEA). Eur J Nucl Med Mol Imaging (2021) 48(10):3286–302. doi: 10.1007/s00259-021-05450-9 34215923

[8] Sarachana T Hu VW. Differential recruitment of coregulators to the RORA promoter adds another layer of complexity to gene (dys) regulation by sex hormones in autism. Mol Autism (2013) 4(1):39. doi: 10.1186/2040-2392-4-39 24119295 PMC4016566

[9] Gao S Li A Liu F Chen F Williams M Zhang C. NCOA5 haploinsufficiency results in glucose intolerance and subsequent hepatocellular carcinoma. Cancer Cell (2013) 24(6):725–37. doi: 10.1016/j.ccr.2013.11.005 PMC3891053 24332041

[10] Dhar D Seki E Karin M. NCOA5, IL-6, type 2 diabetes, and HCC: the deadly quartet. Cell Metab (2014) 19(1):6–7. doi: 10.1016/j.cmet.2013.12.010 24411937 PMC3947913

[11] Chen GQ Tian H Yue WM Li L Li SH Qi L. NCOA5 low expression correlates with survival in esophageal squamous cell carcinoma. Med Oncol (2014) 31(12):376. doi: 10.1007/s12032-014-0376-y 25416054

[12] Ye XH Huang DP Luo RC. NCOA5 is correlated with progression and prognosis in luminal breast cancer. Biochem Biophys Res Commun (2017) 482(2):253–6. doi: 10.1016/j.bbrc.2016.11.051 27847318

[13] Sun K Wang S He J Xie Y He Y Wang Z. NCOA5 promotes proliferation, migration and invasion of colorectal cancer cells via activation of PI3K/AKT pathway. Oncotarget (2017) 8(64):107932–46. doi: 10.18632/oncotarget.22429 PMC5746116 29296214

[14] Edge SB Compton CC. The American joint committee on cancer: the 7th edition of the AJCC cancer staging manual and the future of TNM. Ann Surg Oncol (2010) 17(6):1471–4. doi: 10.1245/s10434-010-0985-4 20180029

[15] Li SH Tian H Yue WM Li L Li WJ Chen ZT. Overexpression of metastasis-associated protein 1 is significantly correlated with tumor angiogenesis and poor survival in patients with early-stage non-small cell lung cancer. Ann Surg Oncol (2011) 18(7):2048–56. doi: 10.1245/s10434-010-1510-5 21290196

[16] Zheng ZC Wang QX Zhang W Zhang XH Huang DP. A novel tumor suppressor gene NCOA5 is correlated with progression in papillary thyroid carcinoma. Onco Targets Ther (2018) 11:307–11. doi: 10.2147/OTT.S154158 PMC5769572 29391807

[17] Liang Y Zhang T Shi M Zhang S Guo Y Gao J. Low expression of NCOA5 predicts poor prognosis in human cervical cancer and promotes proliferation, migration, and invasion of cervical cancer cell lines by regulating notch3 signaling pathway. J Cell Biochem (2019) 120(4):6237–49. doi: 10.1002/jcb.27911 30335900

[18] Chudecka-Glaz AM. ROMA, an algorithm for ovarian cancer. Clin Chim Acta (2015) 440:143–51. doi: 10.1016/j.cca.2014.11.015 25447706

[19] Tan Y Liu F Xu P. Knockdown of NCOA5 suppresses viability, migration and epithelial-mesenchymal transition, and induces adhesion of breast cancer cells. Oncol Lett (2021) 22(4):694. doi: 10.3892/ol.2021.12955 34457049 PMC8358617

[20] Zhang X Liu G Kang Y Dong Z Qian Q Ma X. N-cadherin expression is associated with acquisition of EMT phenotype and with enhanced invasion in erlotinib-resistant lung cancer cell lines. PloS One (2013) 8(3):e57692. doi: 10.1371/journal.pone.0057692 23520479 PMC3592915

[21] He J Zhang W Li A Chen F Luo R. Knockout of NCOA5 impairs proliferation and migration of hepatocellular carcinoma cells by suppressing epithelial-to-mesenchymal transition. Biochem Biophys Res Commun (2018) 500(2):177–83. doi: 10.1016/j.bbrc.2018.04.017 29626478

[22] Williams M Liu X Zhang Y Reske J Bahal D Gohl TG. NCOA5 deficiency promotes a unique liver protumorigenic microenvironment through p21WAF1/CIP1 overexpression, which is reversed by metformin. Oncogene (2020) 39(19):3821–36. doi: 10.1038/s41388-020-1256-x PMC7210077 32203160

[23] Delgado Bolton RC Calapaquí Terán AK Pellet O Ferrero A Giammarile F. The search for new 2-18F-FDG PET/CT imaging biomarkers in advanced ovarian cancer patients: focus on peritoneal staging for guiding precision medicine and management decisions. Clin Nucl Med (2021) 46(11):906–7. doi: 10.1097/RLU.0000000000003784 34238809

[24] Perelli F Mattei A Scambia G Cavaliere AF. Editorial: methods in gynecological oncology. Front Oncol (2023) 13:1167088. doi: 10.3389/fonc.2023.1167088 36969075 PMC10036035

